# 

**Published:** 1892-03-19

**Authors:** 


					gO2 THE HOSPITAL. Makch 19, 1892.
To Our Readers.
On and from the issue of April 2nd, 1892, The
Hospital -will be permanently enlarged, important
features will be introduced, and the price raised from
?ne Penny to Twopence. Those of our readers who
like to subscribe direct, by sending their subscriptions
to the Publisher, at the Office, 140, Strand, W.C., will be
supplied with The Hospital every week, post free, for
8s. a year; 5s. for half a year; or 2g. 6d. for three
months. All subscriptions will be payable in advance.
ITotices of Births, Deaths, and Marriages, are inserted in
this column at a charge of 2s. 6d. for an announcement not exceeding
four lines.
March 19, 1892. THE HOSPITAL. 303
General Charities.
[This Column is devoted to an account of work of charitable institu-
tions other than Medical Charities. The Editor will be glad to receive
reports or news of work at 140, Strand, Philanthropy should be
plainly written on the envelope.]
The late Mr. Alexander Druce has bequeathed ?100 to'tho Earlswood
Asylum for Idiots, and a similar sum to the British Orphan Asylum.
On her return from the Continent, the Duohess of Teck will perform
the ceremony of opening the new Seamen's Institute at the Millwall
Docks, but the ceremony will probably not take place till July.
A sale of real Irish made work is to be held, by the kind permission of
the Marchioness of Londonderry, at Londonderry House, on behalf of
the Irish Industries Association, Motcomb Street, on the 24th and 25th
?f this month.
Good work eeems to have been done during the past year by the
committee of the Drury Lane Working Girls' House and Day Nursery.
Though the expenses have latterly increased somewhat considerably
??ing;to the gieater number of dinners supplied to factory girls and
?thers, the financial condition of the sooiety is in a satisfactory state.
The educational and sooial branches of the work have been oonducted
with an efficiency and success whioh enables the management to point
110 a year of substantial benefit to the district in whioh they labour.
At the annual general court of the CLZBOY ORPHAN" COR-
PORATION, the Committee announced an inorease during the past
year in the receipts from voluntary sources of ?186. Notwithstanding
this satisfactory element in the position of the Corporation, it appears
th&t the Committee has been compelled to raise ?2,000, in order to meet
estraordinary expenditure. Considerable progress has been made with
the new sanatorium, the laboratory and infirmary have been enlarged,
a^d the drainage of the orphanages, which now contain 125 boys and 87
Suls, has been reconstructed.
Archdeacon Farrar pleads on behalf of the London Police Court
fission of the Church of England Temperance Society, in the hope
those who wish their Lenten self-denial to take the form of
8?iierosity to deserving causes will send contributions to him at 17,
ean's-yard, Westminster, in aid of this work. Sir John Bridge has
8l*en his testimony that " there is no mission or charity in London
Wch does so much good work, or does it at so small an expense." The
^Un of ?3,000 a year is required to carry out the work, which is carried
11 through the aid of " Missionaries " who are stationed at every police-
^^rt in London, and who by timely aid and sympathy are able to rescue
any who have sunk into the lowest depths of misery. Last year this
Um feU short by ?500.
An institution which has earned no small measure of success during
? Past twenty-eierht years, but not more than it deserves, is THE
WORDING MEN'S COLLEGE, 48, Great Ormond Street, W.C.
Rinded by the late P. D. Maurice, the position and prospects of the
llef?e now afford good reasons for congratulation on acoount of the
* accomplished. The students are chiefly working men, and the
0*?hers members ?f the Universities and of the different professions,
oh who have themselves been students in the college. Thus the
aimed at of uniting these classes together by associating them
ia al6 001111,11011 work of teaching and learning is gained. The teaching
Wh' wbolly unpaid, and by providing instruction in subjects with
li^L c~ everyone should be acquainted, an endeavour is made to place a
and e5ucation within reach of working men. The increase of work
-tivity within the walls of the college, though affording good
Pens eg *?r OODStatu1ation, tend naturally to inorease the working ex?
face vi't Unf.ortnilately the subscriptions and donations have not kept
than 1 ^ crease, and those who have the means oannot do better
ribute to tho maintenance, and, we hope, expansion of this
^he a
^ambet^t^^kop [Canterbury, who presided at the conference at
Coxjjri lace of tho:,e interested in the work of the CHILDREN'S
of a BtJ HOLIDAYS FUND. laid great stress on the adoption
^Ork 0jS?6t'on which has frequently bjen made in these oslumns. The
Active f / S03iety won d not only be better known but would be pro-
co-?p ?. ir more t xtensive good, as we have pointed oat, if there were
Waller ^ ^tween thn, the leading society, and the many other
Waller EOc|e^i0a which take the came work in hand. It is for the
pretent B00iet;es to communicate with the principal one. Under the
lie d0E?8^^ m a BCQ 1 deal of injury is, unwittingly of course, likely to
fort&i p" n?t unusual for one child to receive tho benefit of several
has c,q>1 8 holidays lrom different societies, while another ohild who
Sether ?trong a claim as his more fortunate brother is left alto-
state cf?fr the cold" Oo-operation would prevent this nndesirable
fee] E which only leads to justifiable discontent and unpleas-
Power tm?8'a ?tal e of affairs which the smaller societies have in their
gD0(i WoT*"* any injury to themselves. A greit deal of
year Ka is alleady accomplished by the Holidays Fund, whxh last
of va8t J6 conntry t olidays to 25.00J children, but this work is capable
c?"?Ptra*neUBi0U i? the Bmaller societies will give up their rivalries and
Scraps and Cleanings.
The census of New York has been completed, and the population is
officially announced to be 1,800,000.
Dueing the past mcnth no less than ?5,700 has been subscribed
towards the proposed National Hospital for Consumption, Dublin.
Me. Henry Habben has Riven one thousand pounds to the fund for
building a new wing to the North London Hospital for Consumption at
Hampst ad.
The Worshipful Company of Drapers have forwarded ?21, and the
trnstees of Berman's Charity ?10 10s., in aid of the funds of the Royal
Westminster Ophthalmic Hospital,
The second of a series of three dances given in aid of the North.West
London Hospital was a brilliant success, and it is hoped that the charity
will benefit substantially by the proceeds.
The fifty-third annual report of the Royal Berkshire Hospital shows
that 1,285 in-patients and 2,045 out-patients were treated during the
past year. The total receipts amounted to ?8,486, and the expenditure
to ?7,069.
De. Louia Pabkes, in addressing the Sanitary Institute, Regent
Street, stated that, notwithstanding all its sanitary improv ments, the
air and water of London were undergoing a process" of continuous
degeneration.
The annual report of the Hastings and St. Leonards Hospital for 1891
gives the number of patients treated as 3,813, against 2,861 in 1890.
showing an increase of 432. Of these 454 were in-patients, and 2,859
out-patients.
The Nurses' Home opened by Mrs. Horefall last month in connection
with the above Institution ; supplies a much-needed want in providing a
comfortable Home for the nurses when cff duty, and ako accommoda-
tion for the staff.
By command of the Princess of Wales the ?1,000 realised by the sale
of Can n Fleming's memorial sermon on (the death of the Duke ot
Glarenoe, has been divided between the Gordon Boys* Home and the
British Home for Incurables.
Diphtheeia has broken out in tl e district of Saffron Walden, Essex,
six cases bave already been removed to the infectious cieea es hospital.
At iNewport, a small village near, several cases of a severe type are
repor'ed, tome too serious to be lemoved.
The annual report of the Convalescent Home for Children, 10,
Clarendon Road, Southsea, states that 101 children were received during
the p< st year. The receipts amounted to ?1,442 17s. 9d. which after all
exf^nses were paid lefta balance to the Treasurer of ?57 18s. lOd.
De. Immisch, the Heidelberg " duel" doctor, has recently passed
away. For over forty years he occupied the post of doctor to the
students who were wounded in duels. All the students ofjthe Heidelberg
University attended the professor's funeral, each corps bearing .its
banners and devices.
It has been decided to establish a Memorial Fand to the late Sir
Morell Mackenzie, the proceeds of which shall be devoted to some per-
manent addition to the Throat Hospital, Golden Square, an institution
founded by Sir Morell, and to which, for a period of twenty-seven years,
he devoted the greater part of his time.
The Royal Southern Hospital, Liverpool, was benefited by Hospital
Saturday and Sunday collections to the amount of ?1 728. Important
sanitary improvements have been carried out daring the past year, and
the operating theatre has been restored to meet its present require-
ments. ?2,000 has been expended upon the work.
It is calculated that 40,000 tons of coal are burnt daily through the
winter in the metropolis, from which a daily quantity of 200 tons
escapes into the air as fine carbon or soot, with probably an equal
amount of sulphur as sulphurous acid?60,COO tons of carbonic gas assist
also in vitiating the atmosphere. Can London fogs be wondered at ?
The B it hop of Marlborough presided last week at the third monthly
meetirg of the Funeral Reform Association. It was stated that Lord
Salisbury had replied to the memorial of the association that attention
to improved burial legislation was impossible this session. It was ie-
solved to present a petition to Parliament on behalf o! the association.
A hew cottage hospital is about to be built at Willesden, from the
designs of Messrs. Newman and Newman, of 81, Tooley Street. London
Bridge. The accommodation to be at first provided is for nine beds,
with the necessary offices ; bnt the plans provide for the future exten-
sion of the buildin? to acoommodate eight males and eight females, with
a separation ward.
At the meeting of the Governors of the New Hospital for Women.
Euston Road, the Rev. Llewellyyn Davies, Chairman, the annualreporr,
showed a steady increase in the numbers of botu the in patients and
out-patients treated, entailing constantly greater expenses, and the hope
was ex Teased by tho Governors that this would be considered by sub-
scribers and the publio.
In the annual report of the Pea body Trust Fund the trustees state
that the net gain of the past , year from rentp and interest amount*
to ??9,695. The capital expenditure on land and buildings was
?1 23.3,404. The artisan ard labouring poor of LoLdonh .ve been pro-
y.d'ed with 11.273 rooms, besides bath-rooms, laundries, and wash-houses,
occupied by 20,269 persons.
The annual meeting of the Royal Albert Orphan Asylum, Bagshot,
wav held on the 9th inst., Colonel Hon. Charles Eliot being in the chair.
Owing to the festival dinner presided over by H.R.H. the late Duke ot
Clarence and Avondale, the Committee have beau able to reduce the
heavy liabilities on the asylum while a reduction has been made in the
m> tazement. A special fuid has been opened for the crecdon of an
infi mary ward.
DssriTE the low temperature of last week, which was nearly seven
degre s below the average of the last twenty year?, the health of London
was good, thfl mortality being only at the rate of 18*9 p<r 1,000 per
ar nuai. Ouly five deaths were ascribed to inflaetza, and the fatal cases
of chest diseasa were considerably under the average The death-iato
of Brighton was strangely higher than the metropolis being 24'2,
March 19,1892.
THE HOSPITAL.
The Student of Medicine.
APPOINTMENTS.
W. H. Barrett, M.B., appointed Lecturer on Pathology at
Queen's College, Belfast. W. E. Bennett, M.R.C.S., L.R.C.P.,
Lond., appointed Senior Medical Officer Cornwall Works
Dispensary, Smethwick, Birmingham, vice T. H. Underbill,
M.B., resigned. Frances May Dickinson Berry, M.B.,
Lond., appointed Assistant Physician for Out-patients
to the New Hospital for Women. J. Hazlewood Clay-
ton, M.B., M.R.C.S., appointed Casualty Surgeon to the
Queen's Hospital, Birmingham, vice Augustus Clay, re-
signed. J. G. Clegg, L.R.C.P. Lond., M.B.C.S., appointed
House-Surgeon to the Manchester Royal Infirmary. W. R.
Cossham, M.D. C.M.Aberd., M.B.C.S., reappointed Surgeon to the
Cirencester Cottage Hospital. E. C. Cripps, L.B.C.P.Lond.,
M.R.C.S. reappointed Medical Officer to the Cirencester Cottage
Hospital. H. P. Dean, M.B.Lond., F.R.C.S., L.S.A., appointed
Assistant Surgeon to the London Hospital, vice H. A. Reeves,
F.R.C.S., resigned. George M. Edmond, M.A., M.D.Aberd.,
reappointed Physician to the Aberdeen General Dispensary. 0.
H. Fowler, M.R.C.S., reappointed Senior Surgeon to the
Cirencester Cottage Hospital. John Gordon, M.D., M.B. C.M.
Aberd., reappointed Physician to the Aberdeen General Dis-
pensary. Arthur George Haydon, L.R.C.P.,Lond., M.R.C.S.
Eng., L.S.A.Lond., appointed Junior House-Surgeon to the
London Temperance Hospital. Herbert Horrocks, M.B., B.sc.
Lond., L.R.C.P., M.R.C.S., appointed House-Physician to the
Hospital for Consumption and Diseases of the Chest, Brompton.
Dudley C. Johnston, L.RC.P., M.R.C.S., appointed House-
Physician to Charing Cross Hospital. C. Mackinnon, M.B.,
C.M.Gla3., reappointed Medical Officer to the Cirencester Cottage
Hospital. Robert D. Muir, M.R.C.S, L.R.C.P., appointed
House-Surgeon to Charing Cross Hospital. J. E. Piatt, M.D.
Lond., L.R.C.P., M.R.C.S., appointed Resident Medical Officer
to the Barnes Convalescent Hospital, Cheadle, Manchester. W.
B. Pritchard, L.R.C.P.,Lond., M.R.C.S., appointed House-
Surgeon to the Manchester Eoval Infirmary. J. Ferguson
Ruxton, M.B., C.M.Aberd., reappointed Physician to the
Aberdeen General Dispensary. Frederick John Smith, B.A.
Oxon., M.D., M.B., M.R.C.P.Lond., F.R.C.S.Eng., appointed
Consulting Physician to the City of London and East London
Dispensary. Edgar Stevenson, M.B., C.M.Aberd., appointed
House-Surgeon to the Liverpool Eye and Ear Infirmary. John
Dixon Stubbs, M.B., B.C.Camb., (Trin. Coll.) appointed House-
Surgeon to the London Temperance Hospital. J. Henry
Williamson, M.R.C.S., L.R.C.P., Lond., appointed Junior
House-Surgeon to the Ancoats Hospital.
VACANCIES.
Resident Medical Officers.
The following vacancies are announced this week, the amount of
salary in each oaee being given after the name of the institution :
Bedford Gene-al Infirm ry, Surgeon, donbly qualified?Salary ?100, &o.
Cardiff Infirmary, Assistant House-Snrgeon. Residence, &c.
City of Liverpool Infectious Diseases Hospitals, Melical Officers for the
City Hospital North, Netherfield Road, and Oity Hospital South,
Grafton Street, doubly qualifid, and not more than 3D years of age
?Salary ?100 per annum, increasing ?10 annually to ?120, &c.
Exeter City Asylum, Digbys, nearjExeter.lAssistant Medical Officer, un-
married?Salary ?100, &a.,risinar ?10 annually to ?150.
Monkwearocouth and South wick Hospital, House Surgeon, doubly
qualified, unmarried?Salary ?80,&c.
Seamen's Hospital Society, S.E., Junior House Surgeon for Branch
Hospital, Royal Victoria and Albert Docks, E., doubly qualified-
Salary ?60, &o.
iron-Resident Medical Officers.
London Lock Hospital and Asylum, Harrow Road, W? and 91, Dean
Street, Soho, W ?Registrar.
National Orthopedic Hospital, 234, Great Portland S'reet, W.?
Registrar. Honorarium, ?20 per annum.
West End Hospital fo- Diseases of the Nervous System, Paralysis, and
Epilepsy, 73, "VVelbeck Street, W., three Physicians, one Surgeon,
one Surgeon for Throat and Ear, one Ophthalmio Surgeon, and one
Dentil Surgeon.
THE HOSPITAL.
March 19, 1892.
THE STUDENT OF
NOTES PEOM THE SCHOOLS.
Cambridge University.
Examinations.?The Registrary publishes the following dates
for the medical examinations in the Easter Term:?
Eirst M.B.
Part I ..
Part II..
Second M.B.
Part I ..
BP" Part II..
Third M.B.
Parts I and II
M.C
Names
sent in.
May 21st
a
>> >>
>? >>
April 9th
Certificates
Received.
June 2nd
? 3rd
? 8th
? 2nd
April 20 th
,, 25 th
Examination
Begins.
June 7th
? 9th
? 13th
? 7th
April 26th
? 29th
Guy's Hospital.
The current number of the Hospital Gazette contains notes on
a clinical lecture on " Tubercular Meningitis," by Dr. Goodhart.
An excellent map of the Eastern Charity District accompanies the
present issue.
St. Mary's Hospital.
The annual "Sports" dinner was held at the Holborn
Restaurant, on Tuesday, the 8th inst. Thesre was a large gather-
ing of past and present students of the hospital. The chair was
occupied by Mr. Field.
PASS LISTS.
Cambridge University.
MxrtrcAL Degrees.?At the last congregation the following decrees
were conferred: M.B. and B.C.?Sydney Kent, B.A., Trinity; John
Dixon Stubba, M.A., Trinity; George Armstrong Mason, M.A., St.
John's; M. R Phipps Dorman, B.A., Clare; W. G. Hawkins Bradford,
B.A., Cains; W. H. Cantrell tehaw, M.A., Jesus; F. Gore Wallace,
B.A,, non-collegiate.
Acts.?Herbert E. Durham, M.A., King's, and J. B. Gillam, B.A,,
Downing, have kept the Acts as required for the degree of Bachelor of
Medicine.
Koyal College of Physicians.
An extraordinary Gomitia of the College was held on Thursday. March
3, 1392, Sir Andrew Cltwk, Bart., President, in the ohair. [The licence of
MEDICINE?(continued),
the College was gTanted to A. R. Mackinnon. Aberdeen ; M. A. Rsid?
Edinburgh ; R. M. Smyth, St. Mary's ; E. H. E. Stack, St. Bartholo-
mew's.
Koyal College of Surgeons of England.
The following gentlemen, having passed the necessary examinations
and having conformed to the by-laws and regulations, were, at an
ordinary meeting of the Council .on March 10th, admitted members of
the College: G. B. Howe.LS.A., Oweni College and Royal Infirmary
Manchester; J. Proaser-Evana, L.R.O.P.Lond., University College.
ATHLETICS.
Football.
Association Cup.?The final tie between St. Bartholomew's and St.*
Mary's was played at Leyton, on Wednesday, March 9th, and after a
keenly contested game, resulted in a draw?one goal each.
Streatham v. St. Bartholomew's Hospital ?Played on Saturday
last, and ended in a win for St. Bartholomew's by six goals to five.
Rugby.
Inter-Hospital Challenge Cup.
St. Thomas's v. St. Bartholomew's.?This match was played at
Richmond on Monday, March 14th. Very little interest was displayed
in the contest, which was a very one-sided affair. At half time St.
Thomas's led by two goals and three tries to nothing, and tbey ultimately
won by twogoals and eight triea (23poiats) to nothing. The place kick-
ing was exceedingly faulty. The following were the teams : St. Thomas 0 '?
V. Graham (back), E. Bromet, W. H. Thorman, and J. L. Prain (three-
quarter backs), N. Hood and A. Montague (half-back*), F.W.J. Good*
hue (captain), J. Carver, R. W. Ord, R. L. Storrar, H. Haward, H>
Knight, P. Blaber, E. SntcliiT, and W. Ashford (forwards). St. Bar-
tholomew's : H. Bond (baok). W N. Soden. J. E. G . Calverley, and
Beath (three-quarter backs), A. Kennington and T. Martin (half-backs)?
G.G.Oakley (captain), E.S.Humphrey, J. W. W. Stephens, P. "?
James, H. B. Meakin, J. C. A. Dunn, W. K. Mercer, H. G. C. Dring.and
E. S. Cardell (forwards). Touch Judges: Mr. W. T. Milton (St?
Thomas's) and Mr. A. N. Weir (St. Bartholomew's). Referee: Mr.
W. Senior (St. Thomas's Hospital and Blackheath).
Winners or the Cup :?
1874-5?Guy's
1875 6?St. George's
1876-7?Guy's
1877-8?St. Thomas's
1878-9?Guy's
1879 80?St. George's
1880-1?St. Bartholomew's
1881-2?St. George's
1882-3?St. Bartholomew's
lf-83-4?London
1884 5?London
1885-6?Guy's
18 S6-7?Middlesex
1887-8?St. Thomas's
1888-9?St. TbomaB's
1889-90?St. Thomas's
1890-1 - Undecided
18J1-2?St. Thomas's

				

